# Quantification of tumour evolution and heterogeneity via Bayesian epiallele detection

**DOI:** 10.1186/s12859-017-1753-2

**Published:** 2017-07-25

**Authors:** James E. Barrett, Andrew Feber, Javier Herrero, Miljana Tanic, Gareth A. Wilson, Charles Swanton, Stephan Beck

**Affiliations:** 1 UCL Cancer Institute, University College London, London, UK; 2 The Francis Crick Institute, London, UK; 3 Cancer Research U.K. Lung Cancer Centre of Excellence, UCL Cancer Institute, London, UK; 4 University College London Hospitals NHS Foundation Trust, London, UK

**Keywords:** Epigenetics, Phylogenetics, Heterogeneity

## Abstract

**Background:**

Epigenetic heterogeneity within a tumour can play an important role in tumour evolution and the emergence of resistance to treatment. It is increasingly recognised that the study of DNA methylation (DNAm) patterns along the genome – so-called ‘epialleles’ – offers greater insight into epigenetic dynamics than conventional analyses which examine DNAm marks individually.

**Results:**

We have developed a Bayesian model to infer which epialleles are present in multiple regions of the same tumour. We apply our method to reduced representation bisulfite sequencing (RRBS) data from multiple regions of one lung cancer tumour and a matched normal sample. The model borrows information from all tumour regions to leverage greater statistical power. The total number of epialleles, the epiallele DNAm patterns, and a noise hyperparameter are all automatically inferred from the data. Uncertainty as to which epiallele an observed sequencing read originated from is explicitly incorporated by marginalising over the appropriate posterior densities. The degree to which tumour samples are contaminated with normal tissue can be estimated and corrected for. By tracing the distribution of epialleles throughout the tumour we can infer the phylogenetic history of the tumour, identify epialleles that differ between normal and cancer tissue, and define a measure of global epigenetic disorder.

**Conclusions:**

Detection and comparison of epialleles within multiple tumour regions enables phylogenetic analyses, identification of differentially expressed epialleles, and provides a measure of epigenetic heterogeneity. R code is available at github.com/james-e-barrett.

**Electronic supplementary material:**

The online version of this article (doi:10.1186/s12859-017-1753-2) contains supplementary material, which is available to authorized users.

## Background

Epigenetic variability allows greater phenotypic diversity and plasticity within a population of genetically similar cells. Epigenetic diversity within a tumour provides a mechanism for clonal evolution and the emergence of resistance to therapy [1]. Persistence of treatment-resistant subclonal populations may explain the failure of some therapies, and higher levels of heterogeneity have been associated with poorer clinical outcomes [2]. Analysing multiple tissue samples from different tumour regions facilitates quantification of tumour heterogeneity and phylogenetic analyses. It has been shown that intra-tumour DNAm heterogeneity is predictive of time-to-relapse in diffuse B-cell lymphomas [3], and that both epigenetic and genetic alterations reflect the evolutionary history of prostate cancers [3]. A recent study of Ewing sarcoma also found substantial levels of epigenetic heterogeneity within tumours [4].

Epigenetic modifications play an important role in the regulation of gene expression. One of the most common types is DNA methylation (DNAm) — where a methyl group is added to the fifth carbon of cytosine. We will focus on DNAm in the canonical CpG context where cytosine (C) is followed by guanine (G). High levels of DNAm in promoter regions are associated with suppressed gene expression whereas increased methylation in gene body regions tends to have the opposite effect [5].

Reduced representation bisulfite sequencing (RRBS) is a sequencing technique that measures DNAm [6]. The experimental protocol consists of treating DNA with bisulfite which converts unmethylated cytosines into uracils. During the amplification process uracils are converted into thymines. After sequencing and comparison to a reference genome, unconverted CpGs are identified as unmethylated and vice versa. The RRBS technique does not sequence the entire genome, but rather regions of the genome that are enriched for CpGs. This naturally splits the genome into distinct loci which can be analysed separately.

Conventional analyses of DNAm have focused on the average DNAm level per CpG site. This is obtained by examining all of the sequencing reads which contain a given CpG and simply counting how many times it is methylated. This type of analysis, however, fails to take into account the full methylation pattern at a given locus which can be observed by looking at all contiguous CpGs along a sequencing read. If there are *d* CpG sites on one read then there are 2^*d*^ possible methylation patterns, which are called *epialleles* [7]. Sequencing reads that cover the same *d* CpG sites can be compared, and the frequency of distinct epialleles that are present can be calculated. Since each DNA fragment comes from a different cell (more precisely a different allele) this provides a snapshot of how many distinct cellular subpopulations are present within the sample. The additional information acquired from contiguous CpG sites on sequencing reads is not present using array-based platforms. It is becoming clear that leveraging this extra information offers potential insights into the epigenetic landscape that would otherwise be missed [8–10].

If multiple samples are taken from the same tumour then each sample can be analysed to see which epialleles are present, and in what proportion, at a given locus. By tracing the presence and absence of different epialleles across different regions of the tumour and matched normal tissue it is possible to reconstruct the evolutionary history of the tumour regions, and to probe for significant differences between normal and tumour tissue. Moreover, the diversity of epialleles within the tumour provides a measure of overall epigenetic heterogeneity.

The acquisition of tumour samples may result in a mixture of both tumour and normal tissue. By comparing the expression of epialleles within the tumour samples and matched normal tissue it is possible to estimate the sample purity — the proportion of the sample which is tumour tissue. Furthermore, it is possible to decontaminate the tumour samples by effectively ‘subtracting’ that component of the epiallele profile which can be attributed to the contaminating normal tissue. An analysis of differential epiallele expression and phylogenetics can be conducted after decontamination.

We present a Bayesian statistical model to infer which epialleles are present at a given locus. The model infers the epialleles that are present and which epiallele each observed sequencing read corresponds to. One hyperparameter controls the level of noise in the model (which represents errors due to bisulfite conversion, PCR amplification, and sequencing) and this is also inferred from the data. Finally, the total number of distinct epialleles is inferred. This final step is a model selection problem and we use the Akaike Information Criterion to avoid overfitting the model. The Bayesian approach allows the quantification of uncertainty regarding the model parameters. In particular, there may be some ambiguity as to which epiallele a certain observed read corresponds to (if some epialleles are very similar to each other for instance). This uncertainty is incorporated into the epiallele distribution by averaging over the appropriate model parameters with respect to the corresponding posterior density.

### Related work

The additional information garnered from adjacent CpGs can be used to define a measure of variability or heterogeneity within a biological sample. The concept of ‘epipolymorphism’, for instance, has been proposed by [11]. The authors in [12] define a measure of ‘methylation entropy’ based on the Shannon entropy and the authors in [2] developed the concept of ‘proportion of discordant reads’.

The term *allele-specific methylation* has also been used to refer to epialleles. Statistical models have been developed by [13–15] to identify epialleles at a given locus and which epiallele each observed read originated from. These models can infer multiple epialleles but in applications only two epialleles have been assumed. An algorithm to estimate tumour purity and deconvolve the epigenomes of tumour and normal tissue uses a very similar statistical model [16].

The authors of [8] compare the epiallele distribution at two disease stages using a ‘composition entropy difference calculation’. They identify loci with substantial shifts in epiallele composition. They confine their analysis to epialleles defined by four CpG sites. Lee et al. [17] used multinomial logistic regression to test for differences in the epiallele distribution between normal and cancer cells. They report performance that is very similar to the method of [8], but do not constrain their approach to four CpGs. In both of these approaches the epialleles are identified from the raw sequencing data, without any inference step to account for experimental noise.

The authors of [9] develop a statistical model that explicitly takes into account measurement noise due to bisulfite conversion efficiency and sequencing errors. The model allows identification of ‘spurious’ epialleles that are due to measurement error (spurious epialleles will tend to have low counts and be very similar to a dominant epiallele). Noise parameters are manually estimated from experimental data, and missing data are not facilitated by their model.

In summary, an adequate epiallele analysis of DNAm sequencing data should have the following features. It should answer the basic research question of whether there is a difference in the epiallele composition between two or more groups of samples — and identify the loci at which there are significant differences. Ideally, some measures need to be taken to avoid spurious epiallele detection due to experimental noise. In addition, an analysis method will generally need to accommodate variable sequencing depth per loci, a variable number of contiguous CpGs per sequencing read, and missing data. Missing data can arise from partially overlapping reads or gaps in a read due to non-overlapping paired-end sequencing protocols.

In addition to the above features, our Bayesian approach automatically infers all model parameters (including the total number of epialleles) from the observed data. Ambiguity in model parameters is explicitly incorporated in our analysis by averaging over the appropriate Bayesian posterior density (descried in detail below). We have applied our method to data from multiple tumour regions and matched normal tissue. We have developed a protocol for estimating the tumour sample purity and consequently decontaminating the inferred epiallele profiles. Although we have focused on multi-region tumour sampling our method could be applied to a single sample also.

## Methods

Sequencing reads are aligned to the reference genome and organised into different genomic *loci*. A locus is a region of the genome containing *d* CpG sites (*d* can take different values to each locus). Due to the nature of RRBS data the sequencing reads naturally tend to form non-overlapping loci. In our paired-end experimental protocol up to 125 bp was sequenced at each end of the DNA fragment. It is possible for loci to exceed 250 bp in length if the DNA fragments were longer than this or if multiple reads partially overlapped. Some additional steps were taken to modify loci in order to control the amount of missing data per locus. See Additional file 1 A for full details.

**Fig. 1:**
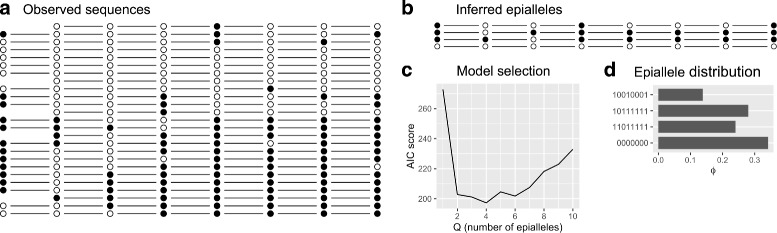
**a** An example of a genomic locus (chr1:1,145,478-1,145,614) in which each row corresponds to a sequencing read. *Black* and *white circles* represent methylated and unmethylated CpGs respectively. Note that some CpG measurements are missing. **b** The four epialleles that are inferred from the observed sequencing reads. **c** The Akaike Information Criterion score versus the total number of epialleles. The inferred number of epialleles corresponds to the minimum AIC score. **d** The proportion of observed reads attributed to each epiallele after marginalisation over the parameter ***w*** (see main text for details)

Let *N* denote the number of sequencing reads at a given locus. To keep our notation compact we will avoid indexing each locus and what follows here is applicable to any locus of the genome. A sequencing read is represented by a *d*-dimensional vector **y**_*i*_∈{0,1}^*d*^ where *i*=1,…,*N* and 0 and 1 correspond to unmethylated and methylated CpG sites respectively. An example is plotted in Fig. 1(a). It is assumed that each observed read can be attributed to one of *Q* epialleles **x**_*q*_ with *q*=1,…,*Q* and *Q*≤*N*. The parameter *w*_*i*_∈(1,…,*Q*) specifies which epiallele read **y**_*i*_ originated from. The observed methylation status of each CpG may differ from the corresponding epiallele status with probability *ε*∈ [0,1/2]. Supposing *w*_*i*_=*q* we can therefore write $p(\mathbf {y}_{i}|\mathbf {x}_{q},\epsilon,Q)=\prod _{\mu =1}^{d} p(y_{i\mu }|x_{q\mu },\epsilon,Q)$ where

1 $$ p\left(y_{i\mu}|x_{q\mu},\epsilon,Q\right)=\left\{ \begin{array}{ll} \epsilon & \quad\text{if \(y_{i\mu}\neq x_{q\mu}\)}\\ 1-\epsilon & \quad\text{if \(y_{i\mu}= x_{q\mu}\).} \end{array} \right.  $$

The epialleles are analogous to latent variables in a latent variable model. Our goal is to infer the quantities **X**=(**x**_1_,…,**x**_*Q*_) and **w**=(*w*_1_,…,*w*_*N*_) as well as the hyperparameter *ε* and the number of epialleles *Q* from the observed data **Y**=(**y**_1_,…,**y**_*N*_). Using Bayes’ theorem the posterior over the unknown quantities is

2 $$ p\left(\mathbf{X},\mathbf{w}, \epsilon|\mathbf{Y},Q\right) = \frac{p\left(\mathbf{Y}|\mathbf{X},\mathbf{w},\epsilon,Q\right)p(\mathbf{X}|Q)p(\mathbf{w}|Q)}{p(\mathbf{Y}|Q)}  $$

where the likelihood is

3 $$\begin{array}{*{20}l} p\left(\mathbf{Y}|\mathbf{X},\mathbf{w},\epsilon,Q\right) &=\prod_{i=1}^{N}\sum\limits_{q=1}^{Q} \delta_{q,w_{i}}\,p\left(\mathbf{y}_{i}|\mathbf{x}_{q},\epsilon,Q\right). \end{array} $$

The delta function is defined by *δ*_*xy*_=1 if *x*=*y* and *δ*_*xy*_=0 otherwise. The marginal density $p(\mathbf {Y}|Q)=\sum _{\mathbf {X}}'\sum _{\mathbf {w}}'\int \mathrm {d}\epsilon '\,p\left (\mathbf {Y}|\mathbf {X},\mathbf {w},\epsilon,Q\right)p(\mathbf {X}|Q)p(\mathbf {w}|Q)$ serves to normalise the posterior density where the summation is over all possible values of **X** and **w**. We will use maximum entropy priors which are uniform densities over the 2^*Qd*^ possible epiallele configurations **X** and *Q* ^*N*^ possible values of **w**.

### Bayesian inference

For fixed **X**,*ε*, and *Q*, the maximum a posteriori (MAP) estimate for **w** is given by attributing each read **y**_*i*_ to the epiallele that is most similar to it. That is,

4 $$ w_{i}^{*} = \text{argmax}_{q} p\left(\mathbf{y}_{i}|\mathbf{x}_{q},\epsilon,Q\right).  $$

Next we wish to obtain the MAP estimate for *x*_*qμ*_ for fixed **w**,*ε* and *Q*. Let *N*_1_ denote the total number of methylated CpGs at site *μ* in observed reads that have been attributed to epiallele *q*. That is, $N_{1} = \sum _{i} y_{i\mu }$ where the sum is restricted to indices for which *w*_*i*_=*q*. Similarly, *N*_0_ is the total number of unmethylated CpGs at site *μ* in reads stemming from epiallele *q*. It is straightforward to show that the MAP estimate is

5 $$\begin{array}{*{20}l} x^{*}_{q\mu} &= 1\quad\text{if \(N_{1} > N_{0}\)}\\ x^{*}_{q\mu} &= 0\quad\text{otherwise}. \end{array} $$

An example is given in Fig. 1(b). We now define the total *matches* at a given locus as $\alpha _{1} = {\sum \nolimits }_{i,\mu } \delta _{y_{i\mu },x_{w_{i}\mu }}$ and *mismatches* as $\alpha _{0} = {\sum \nolimits }_{i,\mu } 1-\delta _{y_{i\mu },x_{w_{i}\mu }}$. It can be shown (see Additional file 1) that the MAP estimate for *ε* is

6 $$ \epsilon^{*} = \frac{\alpha_{0}}{\alpha_{0}+\alpha_{1}}  $$

which is simply the proportion of observed CpGs at that locus that differ from the underlying epialleles. Some values of *y*_*iμ*_ may be missing and these are handled by simply omitting them from sums and products over *i* and *μ*.

#### Algorithm

Note that the MAP estimates **w** ^∗^ and **X** ^∗^ are independent of *ε*. Given a set of observed data **Y** the first task is to determine optimal values for **w** and **X**. This is done according to the following algorithm:

1. Initialise **w** by using hierarchical clustering to group the observed reads **Y** into *Q* groups. The *hamming distance* (the proportion of CpGs that differ between two sequencing reads) is used as a distance measure.
2. Compute **X** according to (5) using the current estimate of **w**.
3. Compute **w** according to (4) using the current estimate of **X**.
4. Repeat steps 2 and 3 until **w** and **X** converge to a steady solution (typically two or three iterations).

Denote the final parameter values as $\hat {\mathbf {w}}$ and $\hat {\mathbf {X}}$. The value for $\hat {\epsilon }$ is then given by (6).

#### Model selection

In principle, the marginal density *p*(**Y**|*Q*) could be used to compare models with different values of *Q*. In practice, however, *p*(**Y**|*Q*) is analytically intractable. Instead we use the Akaike information criterion (AIC) [18] in order to select the optimal number of epialleles

7 $$ \text{AIC}(Q)=-2 \log p\left(\mathbf{Y}\left|\hat{\mathbf{X}},\hat{\mathbf{w}},\hat{\epsilon},Q\right.\right)+2Qd  $$

where $\hat {Q}=\text {argmin}_{Q} \text {AIC}(Q)$. For a model with *Q* epialleles the *Qd* parameters that make up the matrix **X** are regarded as free parameters. The term 2*Q* *d* penalises more complex models (i.e. models with larger *Q*). A more complex model will only be selected if the evidence from the data is sufficiently strong to overcome the penalty term. An example of the AIC score is plotted in Fig. 1(c).

#### Marginalisation of **w**

Finally, it may not be completely clear which epiallele an observed read should be attributed to (there could be several epialleles an equal edit distance away). This ambiguity manifests itself as the uncertainty surrounding the parameter *w*_*i*_. The Bayesian approach allows this uncertainty to be incorporated into our analysis. The marginal density over *w*_*i*_ is given by fixing all other parameters to their MAP values

8 $$\begin{array}{@{}rcl@{}} &&p\left(w_{i}\left|\hat{\mathbf{w}}_{-i},\hat{\mathbf{X}},\hat{\epsilon},\hat{Q} \right.\right)  \\ &=& \frac{p\left(\mathbf{Y}\left|\hat{\mathbf{X}},\hat{\mathbf{w}}_{-i},w_{i},\hat{\epsilon},\hat{Q} \right.\right)p\left(\hat{\mathbf{X}}\left|\hat{Q}\right.\right)p\left(\hat{\mathbf{w}}\left|\hat{Q}\right.\right)}{p\left(\mathbf{Y}\left|\hat{Q}\right.\right)} \end{array} $$

where $\hat {\mathbf {w}}_{-i}$ is a (*d*−1)-dimensional vector obtained from $\hat {\mathbf {w}}$ by removing element *i*. At the given locus in question the *proportion of observed reads originating from epiallele q* is given by

9 $$ \phi_{q}=\frac{1}{N}\sum\limits_{i=1}^{N} p\left(w_{i}=q\left|\hat{\mathbf{w}}_{-i},\hat{\mathbf{X}},\hat{\epsilon},\hat{Q}\right.\right).  $$

The quantity $\boldsymbol {\phi }=(\phi _{1},\ldots,\phi _{\hat {Q}})$ specifies the distribution of epialleles within that locus. An example of ***ϕ*** is given in Fig. 1(d).

### Application to multi-region tumour sampling

We will now describe our analysis protocol. In our application we are considering sequencing data from multiple regions of the same tumour. The number of distinct epialleles present at a particular locus is determined by pooling sequencing reads from all tissue samples (tumour and normal) in order to boost statistical power. Suppose there are *s*=1,…,*S* tumour samples with *N*_*s*_ reads per sample (at a given locus). The total number of reads in the pool is now $N=\sum _{s} N_{s}$. Using the pooled reads a model is fitted as described above. The vector $\hat {\mathbf {w}}$ defines which epiallele each sequencing read originated from. The distribution of epialleles within region *s* is given by

10 $$ \phi_{q}^{s} = \frac{1}{N_{s}}\sum_{i\in I_{s}} p\left(w_{i}=q\left|\hat{\mathbf{w}}_{-i},\hat{\mathbf{X}},\hat{\epsilon},\hat{Q}\right.\right)  $$

where *I*_*s*_ is the set of indices of reads belonging to sample *s*. The vectors ***ϕ*** ^*s*^ serve to characterise each sample in terms of their epiallele distributions.

#### Estimation of sample purity

Suppose $\hat {Q}$ epialleles are inferred at a particular locus of a particular tumour sample (for the sake of compactness we will not index the loci or samples). The locus is characterised by ***ϕ***, the inferred probability distribution over the $\hat {Q}$ epialleles. If the tumour sample is contaminated with normal tissue then we can write

11 $$ \boldsymbol{\phi} = \rho \mathbf{t}+(1-\rho)\mathbf{n}  $$

where *ρ*∈ [0,1] is the proportion of observed tissue that comes from the tumour (the sample ‘purity’), and **t** and **n** are the epiallele distributions in the tumour and normal tissues respectively (at the particular locus in question). For example, if we infer $\hat {Q}=3$ epialleles at a locus and **n**=(0.7,0.2,0.1) and **t**=(0.2,0.2,0.6) then for a purity of *ρ*=0.8 we would expect to observe ***ϕ***=(0.3,0.2,0.5). We can estimate ***ϕ*** and **n** from the observed data at a particular locus. Estimation of both *ρ* and **t** requires solving the $\hat {Q}$ equations in (11) for $\hat {Q}+1$ variables which generally is not possible. However, the quantity

12 $$ \xi=\frac{1}{2}\sum\limits_{q=1}^{\hat{Q}} \text{abs}\left(\phi_{q}-n_{q}\right)  $$

can be computed at each locus of the observed tissue sample. The index *q* sums over all of the epialleles inferred at this locus and *ξ* will take different values at different loci. We can loosely interpret *ξ* as *the proportion of reads unattributable to normal tissue*, and in the example above *ξ*=0.4. If we substitute (11) into (12) we can see that *ξ* takes a minimum value of 0 when **t**=**n**. At a locus in which the tumour and normal tissues have a completely different epiallele composition then we say that if *t*_*q*_>0 then *n*_*q*_=0 and if *n*_*q*_>0 then *t*_*q*_=0 for $q=1,\ldots,\hat {Q}$. It is straightforward to show that if this is the case then *ξ*=*ρ* and that this is the maximum value *ξ* can take.

**Fig. 2:**
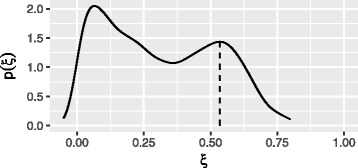
Estimation of tumour sample purity for region 2 of the tumour. The parameter *ξ* was calculated at all eligible loci across the genome and the empirical distribution is plotted here. The sample purity is equal to the maximum value of *ξ* which is interpreted to occur at the rightmost maximum at *ξ*=0.53. The distribution of *ξ* is ‘smoothed’ due to the fact that at each locus *ξ* is estimated from a finite sample of sequencing reads

We therefore expect that *ξ* will take values in the range [0,*ρ*] when computed across all loci of the observed sample. If we plot the empirical density of *ξ* values the parameter *ρ* can be estimated from the maximum value of *ξ*. Since ***ϕ*** and **n** are estimated from finite data samples we expect the distribution of *ξ* to be ‘smoothed’ by sampling noise. This is precisely what we observe in practice. An example of the empirical density of *ξ* is plotted in Fig. 2.

#### Decontamination of normal tissue

Finally, we note that once estimates of *ρ* have been obtained we can calculate the ‘decontaminated’ tumour epiallele profiles at each locus according to

13 $$ \hat{t}_{q}=\frac{\phi_{q}-(1-\rho)n_{q}}{\rho}\quad \text{for}~q=1,\ldots,\hat{Q}.  $$

We have used the notation $\hat {t}_{q}$ to emphasise that this is an estimate of the tumour epiallele distribution. Due to the fact that ***ϕ***, **n** and *ρ* are estimated from finite data samples it is possible that $\hat {t}_{\mu }$ can take values outside [0,1]. Any cases where $\hat {t}_{\mu }<0$ are set to 0 and any cases where $\hat {t}_{\mu }>1$ are set to 1.

A conventional analysis of DNAm sequencing data will typically ‘call’ a methylation level at each CpG site by computing the proportion of reads on which a CpG is observed in a methylated state. Using our method a methylation level for each CpG site can readily be computed after decontamination of normal tissue and used in existing analysis pipelines.

#### Construction of a phylogenetic tree

Using the decontaminated representation of a sample $\hat {\mathbf {t}}_{s}$ the euclidean distance between $\hat {\mathbf {t}}_{s}$ and $\hat {\mathbf {t}}_{s'}$ can be used as a distance measure between samples *s* and *s* ^′^. Each locus provides a distance matrix that depends on the distribution of epialleles at that particular locus. To obtain an overall distance matrix we average over distance matrices from all loci. Any distance based phylogenetic inference method can subsequently be used to construct a phylogenetic tree. We used the ‘fastme.bal’ function as part of the ‘ape’ R package [19].

## Results

### Simulations

Simulations of a single locus were performed to study what effect the number of CpGs, *d*, the number of sequencing reads, *N*, and the noise level, *ε*, have on our ability to correctly detect the underlying epialleles. The simulated reads were noise corrupted versions of three distinct randomly generated epialleles, and on average each epiallele corresponded to one third of the observed reads. To assess model performance we counted the proportion of observed reads that were attributed to their correct underlying epiallele (which requires both inference of the correct epialleles and attribution to the correct epiallele). For every value of the parameters results were averaged over 100 simulations.

We found that *N*=100 and *d*=6 gave a success rate of approximately 95% at a 5% noise level. These values were used to guide the selection of viable loci in subsequent analyses of experimental data. Dropping to *N*=50 gave a performance of just over 90% (Additional file 1: Figure S3). Sequencing depth beyond *N*=100 did not yield any additional performance gain. The performance saturates at 100% for *d*>15 (Additional file 1: Figure S4). Since the number of possible epialleles is 2^*d*^ a larger *d* will typically make it easier to resolve distinct epialleles. Additionally, since the underlying epialleles are randomly generated it is possible that some may be within one edit distance from each other, making it difficult for the model to distinguish between very similar epialleles and noise when *d* is small. Performance was observed to decrease sharply for increasing noise levels (Additional file 1: Figure S5).

### Cell line data: detection of low frequency epialleles

In order to test whether our statistical methods could detect low frequency epialleles in practice we mixed a fully unmethylated and fully methylated cell line in a 9:1 ratio prior to sequencing. Loci with six or more CpGs and 50 or more reads were identified. Within these loci 6.3% of observed CpGs were methylated overall. The two cell lines were sequenced separately and we found that the fully methylated and unmethylated cells were in fact 97.3*%* and 3.8*%* methylated respectively.

The Bayesian model was used to detect the presence of epialleles at each loci. We found that 5.2% of methylated CpGs were attributed to methylated epialleles (defined as epialleles with ≥50*%* methylation). The mean noise level was inferred as 1.1%. This suggests that the majority of methylation is correctly identified as corresponding to a methylated profile and therefore our method is capable of resolving a distinct low frequency cellular subpopulation.

### Multi-region tumour sampling case study

Our case study data consisted of seven tissue samples from a single lung tumour (CRUK0062) along with one matched normal tissue sample. These tissue samples were acquired as part of the larger TRACERx study [20]. The raw sequencing data were trimmed and aligned to a reference genome. Sequencing reads were subsequently organised into distinct genomic loci as described in the Additional file 1. We demanded that no more than 25% of data were missing per locus (due to partially overlapping paired-end reads or reads not covering the whole locus). Any data from chromosomes X and Y were discarded. At each locus $\hat {Q}$ epialleles are inferred and any epialleles that accounted for less than 5% of observed reads were discarded prior to the computation of ***ϕ***_*s*_ for *s*=1,…,*S*. This was done in order to focus on the dominant shifts in epiallele profiles and to minimise the risk of inferring spurious epialleles.

In order to compare the distribution of epialleles within different tumour samples it was necessary to identify all of the loci which occurred in two or more samples. That is, the loci themselves must ‘match up’ between tumour samples in order for a comparison to be made (partially overlapping loci were permitted provided they met the minimum number of non-missing CpG requirements). Only loci with a median read depth ≥100 across normal and tumour tissue samples and six or more CpGs were considered. A total of 39,940 loci were analysed out of which 73% were found to contain a single epiallele, 13% contained two, 7% contained three, 4% contained four, and 3% had five our more (up to a maximum of thirteen).

#### Comparison of epiallele distribution throughout the tumour

**Fig. 3:**
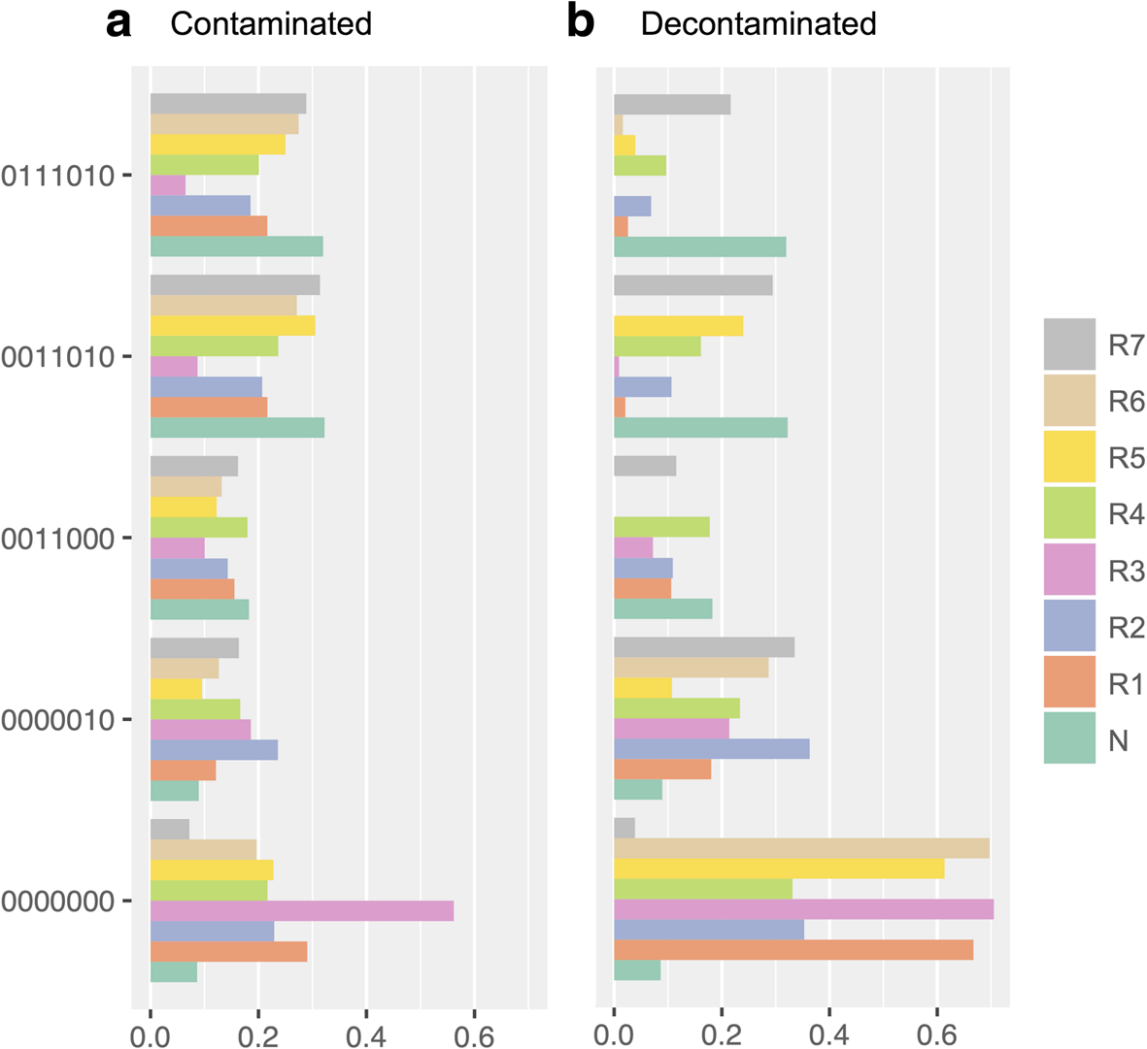
A genomic locus (chr1:2,603,277-2,603,489) composed of seven CpGs. The distribution of five epialleles – inferred using the Bayesian model – are plotted for seven tumour regions (R1 to R7) and one normal sample (N). In **a** the tumour samples have not been corrected for normal tissue contamination whereas in **b** they have been. The tumour samples are shifting towards an unmethylated profile in comparison to the normal tissue. The locus lies in a large intronic region in the gene TTC34

At each locus the Bayesian model is used to infer the epialleles present, the total number of epialleles, and which epialleles each observed sequence came from. An example locus with seven CpGs from chromosome one is presented in Fig. 3. At this locus five distinct epialleles were detected. Both the observed and decontaminated profiles are shown. The normal tissue is predominantly composed of methylated epialleles whereas the tumour samples have a greater proportion of less methylated epialleles. This suggests that within the tumour there exist cellular subpopulations that are undergoing a transition from a methylated state to an unmethylated one.

**Fig. 4:**
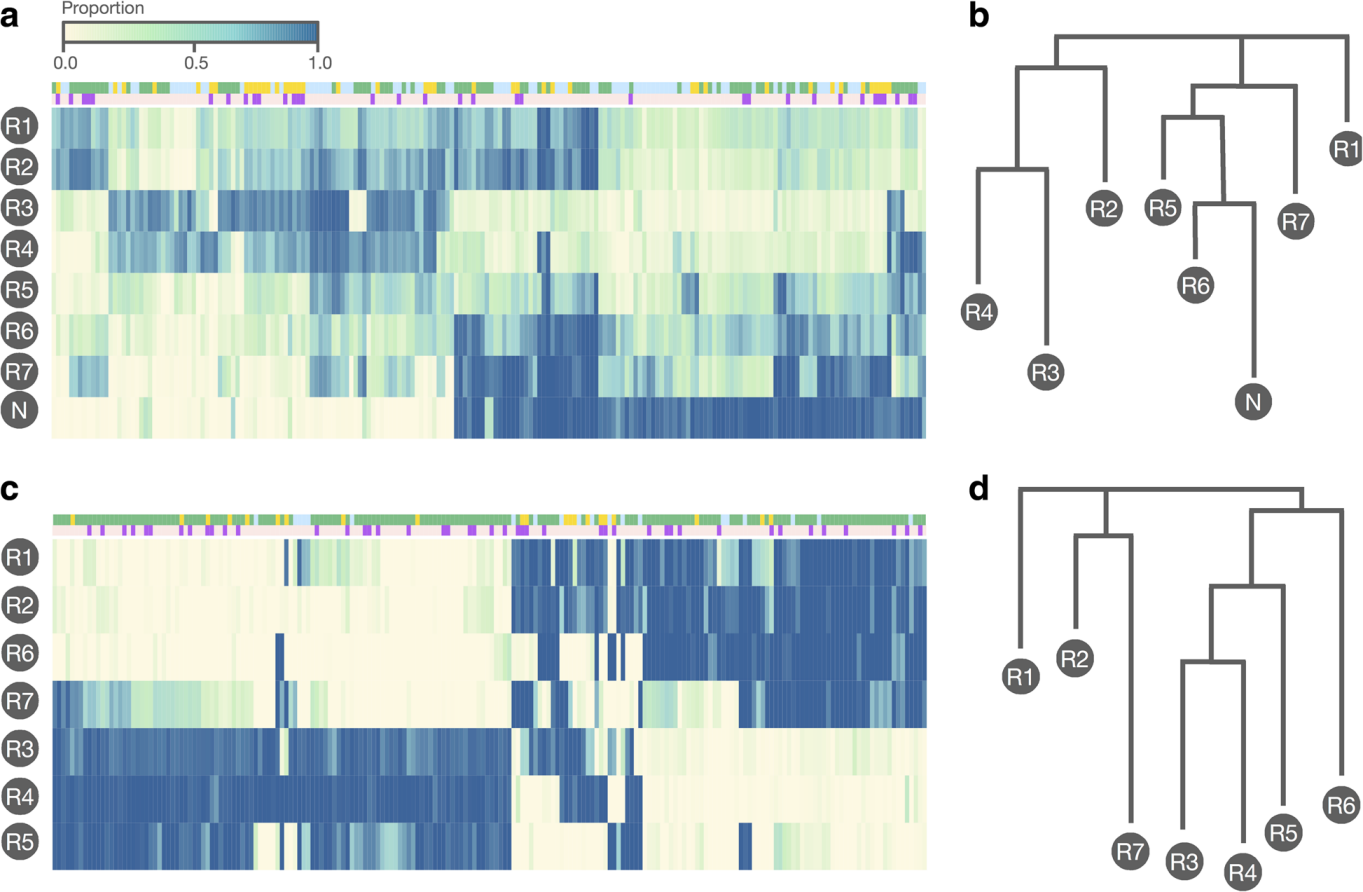
**a** Heatmap of the top 200 most variable epialleles across the seven tumour samples (labelled R1 to R7) and matched normal sample (labelled N). A proportion of 1.0 (*dark blue*) means that that epiallele accounted for all observed methylation patterns at the corresponding locus. These data have not been decontaminated of normal tissue. **b** The phylogenetic tree inferred before correction for contaminating normal tissue. In **c** and **d** are the same figures for the decontaminated epiallele profiles. In the *top* annotation track *green* denotes a CpG island, *yellow* a shore, and *blue* otherwise. In the *bottom* track *dark purple* denotes a gene promoter, otherwise *light pink*. A promoter was defined as between 2kb upstream and 50bp downstream from a transcription start site

In order to understand shifts in epiallele frequency at a global level we plotted a heatmap of the top 200 most variable epialleles in Fig. 4(a) and (c). Both the observed and decontaminated epiallele profiles were used. Tumour samples are characterised by both a loss and gain of numerous epialleles when compared to the normal tissue sample. The variability in epiallele expression throughout different parts of the tumour suggests that a substantial level of tumour heterogeneity exists at the epigenetic level. Note that in the contaminated samples 71 out of the 200 epialleles were located on CpG islands, and 54 were located on a CpG shore (defined as 2 kilobases either side of an island). In the decontaminated version 124 epialleles were located on an island and 38 on a shore.

#### Estimation of sample purity

The sample purities were estimated as described in the methods section. An example of the empirical density of *ξ* within tumour region 2 is plotted in Fig. 2. From the location of the rightmost maximum we estimate *ρ*=0.535. Plots for all tumour regions are given in Additional file 1: Figure S6. Estimates of purity for the seven tumour samples are given in Table 1. For tumour region 6 the rightmost maxima was not visible presumably due to very low tumour purity. The purity estimates are compared to estimates obtained from an analysis of exome data from the same tissue samples performed independently in [20].

**Table 1:** In the middle column are estimates of tumour purity based on a comparison of epiallele distributions between normal tissue and tumour tissue. The third column contains estimates obtained from a separate study of exome data from the same tumour samples

Tumour sample	Epiallele purity estimate	Exome purity estimate
R1	35%	32%
R2	54%	51%
R3	75%	73%
R4	53%	67%
R5	25%	28%
R6	<20%	13%
R7	30%	36%

#### Inference of a phylogenetic tree

Phylogenetic trees were generated as described in the methods section. The trees for both contaminated and decontaminated samples are plotted in Fig. 4(b) and (d). The structure of the contaminated tree is dominated by the sample purities, with low purity samples clustering together. The decontaminated tree has a totally different structure and this is broadly similar to a phylogenetic tree obtained from from a separate genetic analysis of the same patient and shown in Additional file 1: Figure S7.

#### Quantification of epigenetic disorder

**Fig. 5:**
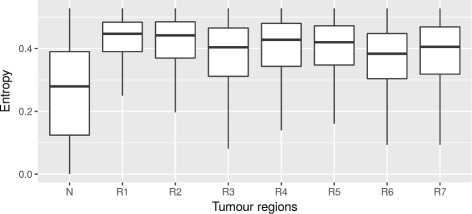
*Box* plots of the Shannon entropy of the epiallele distribution across normal tissue (N) and the seven tumour regions (R1–R7)

The Shannon entropy provides a measure of how disordered a random variable is. In particular, the entropy of the epiallele distribution ***ϕ***_*s*_ quantifies how disordered or heterogeneous each locus is in sample *s*. The epiallele entropy at a given locus is defined as

14 $$ -\frac{1}{d}\sum\limits_{q=1}^{\hat{Q}}\phi_{q}\log_{2}\phi_{q}  $$

where *d* is the number of CpGs at that locus and ***ϕ*** is the inferred probability distribution of epialleles (after discarding low frequency epialleles and marginalisation over the **w** parameter as described above). In Fig. 5 box plots summarise the distribution of entropies across tumour and normal tissues (without decontamination). The tumour tissue samples have a substantially elevated entropy in comparison to the normal tissue. Box plots of the entropies after decontamination of normal tissue are shown in Additional file 1: Figure S8. A comparison to the measures of epigenetic disorder proposed in [2, 11, 12] is presented in the Additional file 1.

## Discussion

Analysis of epialleles allows for a deeper interrogation of the underlying biology than a pointwise examination of CpG methylation states. Tracing the patterns of DNA methylation along epialleles allows one to tease apart different cellular subpopulations and acquire a richer quantification of heterogeneity and disorder that would not be possible by looking at individual CpG sites. In particular, the distribution of epialleles throughout a tumour can shed light on the evolutionary history of the tumour.

Our analysis protocol specifically pools sequencing reads from multiple tissue samples in order to leverage greater statistical power in epiallele detection. Our Bayesian approach will automatically detect the number of epialleles present, and infer what the methylation pattern of those epialleles are. One strength of the Bayesian approach is that it provides a framework for averaging over uncertainty in model parameters. If there is uncertainty as to which epiallele an observed sequencing read may have originated from, then a natural solution is to average over that uncertainty by marginalising over the appropriate posterior distribution. In addition to the above features our model can easily accommodate missing data and can handle an arbitrary sequencing depth and number of CpG sites per locus. Furthermore, by comparing the distribution of epialleles within normal and tumour tissue samples it is possible to estimate the purity of each sample and to subsequently decontaminate them. Methylation levels at each CpG site can be extracted from the decontaminated samples and subsequently used in standard analysis pipelines.

In future work it may be interesting to compare the distribution of loci that are located close to each other. Although it is not possible to phase reads between disjoint loci the number of epialleles and the entropy may be correlated between close loci.

Tracking the presence or absence of epialleles throughout the tumour opens up an additional layer of complexity beyond that of conventional methylation analyses. Pointwise methylation analysis protocols typically average over sequencing reads – to ‘call’ the methylation status at single CpGs – that potentially come from a diverse and heterogenous population of cells. Detecting which epialleles are present allows one to distinguish between these cellular subpopulations and identify tumour subclones that are defined by distinct epialleles. One can then probe changes between normal and cancerous tissue at a finer resolution. As we have demonstrated here, studying epiallele frequencies in different parts of the tumour reveals the evolutionary history of the tumour and allows a phylogenetic tree to be constructed. A measure of disorder or heterogeneity inside the tumour can be obtained through measures such as Shannon’s entropy.

## Conclusion

Understanding tumour heterogeneity is an important step towards understanding why certain therapies fail and why resistance to treatment can emerge. Subclonal populations of treatment-resistant cells can persist after treatment even if they only account for a small fraction of the original tumour. Epigenetic diversity within the tumour may play an important role in tumour evolution alongside genetic variability. It is increasingly recognised that for DNA methylation sequencing data studying the patterns of methylation along the genome – ‘epialleles’ – can provide greater insight into the underlying dynamics of epigenetic regulation than a conventional pointwise analysis.

We have exploited this opportunity to study the distribution of epialleles throughout a tumour by performing reduced representation bisulfite sequencing on seven regions of the same tumour and one matched normal tissue sample. Our new Bayesian approach infers which epialleles are present at a given locus. A comparison of the frequency of different epialleles across the tumour and normal tissue highlights changes between normal and cancerous tissue and allows the extraction of a phylogenetic history. The concept of entropy can be used as a measure of global disorder within the tumour. Our method can be applied more generally to any type of DNAm sequencing data.

Future work will focus on larger scale studies of multiple patients with multi-region tumour sampling in order to probe for systematic alterations in epiallele expression between normal and cancerous tissue. Previously, measures of epigenetic disorder were found to be associated with clinical outcome and it will be interesting to see if quantification of disorder at the level of epialleles will provide a more refined measure of tumour aggressiveness. Ultimately, it is hoped that a clearer elucidation of epigenetic dynamics will complement our genetic knowledge of cancer and provide a more comprehensive understanding of the disease.

## Additional file

Additional file 1 Supplementary figures, results and information. (PDF 1600 kb)
