# Supplementary material for: Quantification of tumour evolution and heterogeneity via Bayesian epiallele detection
Source: BMC Bioinformatics. 2017 Jul 25;18:354. doi: 10.1186/s12859-017-1753-2 (PMC5526259; doi:10.1186/s12859-017-1753-2)
Supplement: Additional file 1 — Supplementary figures, results and information. (PDF 1600 kb) [file 12859_2017_1753_MOESM1_ESM.pdf]

Supplementary material for "Quantification of tumour evolution and heterogeneity via Bayesian epiallele detection"

James E. Barrett*[1], Andrew Feber[1], Javier Herrero[1], Miljana Tanic[1], Gareth Wilson[2,3], Charles Swanton[2,3,4,†], and Stephan Beck[1]

[†] *on behalf of the Lung TRACERx consortium*
[1] *UCL Cancer Institute, University College London, U.K.*
[2] *The Francis Crick Institute, London, U.K.*
[3] *Cancer Research U.K. Lung Cancer Centre of Excellence, UCL Cancer Institute, U.K.*
[4] *University College London Hospitals NHS Foundation Trust, U.K.*

June 2, 2017

A Extraction of viable loci

In Figure 1 is an example of an observed locus before any preprocessing steps have been taken. The locus is defined naturally since it does not overlap with any other observed sequencing reads. Note that several sequencing reads only partially overlap and many do not overlap at all. In addition, several CpG measurements are missing from the middle of some reads due to the paired-end sequencing protocol that was used to generate the data (sometimes the paired ends may not span the full length of the DNA fragment).

Intuitively this locus should be split into two loci as the two blocks of reads overlap by a single CpG which is not enough to phase the inferred epialleles. We implemented the following algorithm to split the observed sequencing reads into sensible loci.

1. Specify the minimum number of contiguous CpGs d_{min} and the minimum number of reads N_{min} required in order for a locus to be admissible (we used $d_{min} = 6$ and $N_{min} = 100$ in practice).
2. Specify the maximum proportion of missing data that is allowed (we used 25% in practice).
3. If the observed locus contains more than 25% missing data move to the next step, otherwise skip to step 4.

   3(a). Represent the observed reads as a matrix with all non-missing measurements equal to 1 and all missing values represented with 0. Use hierarchical clustering to split the reads into two groups using the hamming distance.

*Contact: regmjeb@ucl.ac.uk

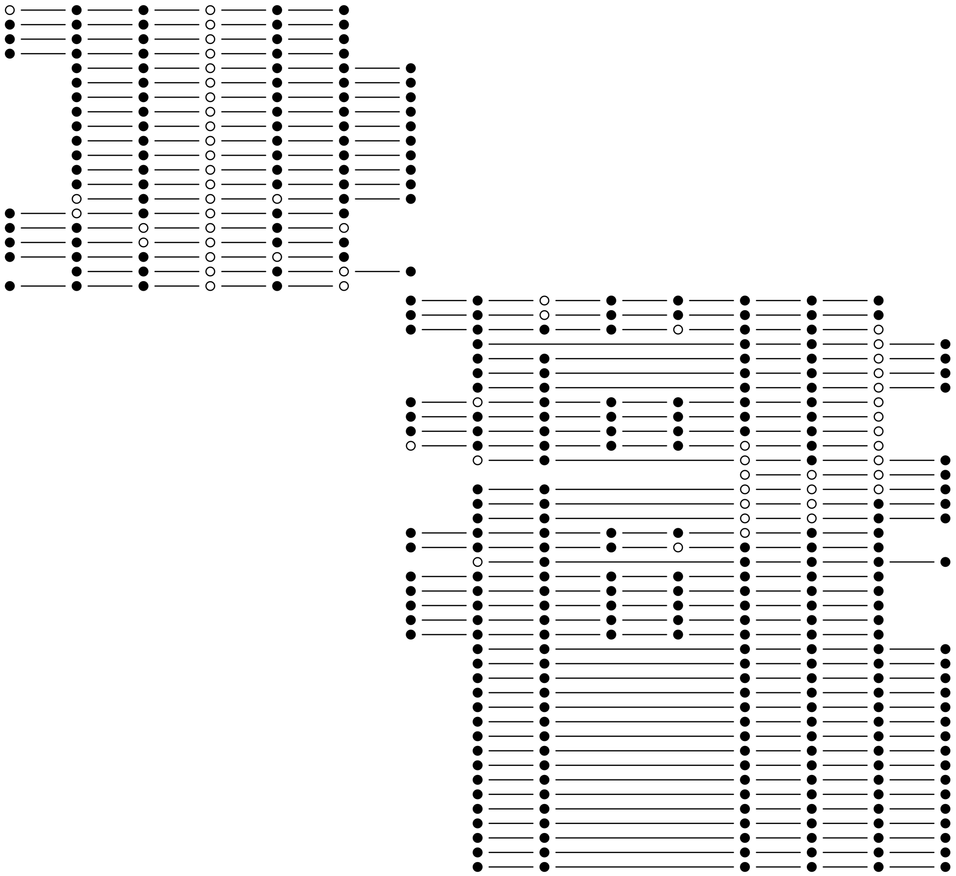

Figure 1: An example of an observed locus (chr1:15,232,224-15,232,587) before preprocessing.

3(b). If the two groups contain less than 25% of the missing data then proceed to the next step. Otherwise repeat the clustering above with three groups and so forth.

4. Discard any loci that fail to meet the minimum values of d and N.

We demanded that, in addition to the loci as a whole, each CpG site should not contain more than 25% missing data. Any CpGs that failed to meet this constraint were discarded. This helped to trim low-coverage CpGs from at the edges of observed loci (for example, the very leftmost CpG in Figure 1). The resulting loci after preprocessing are shown in Figure 2.

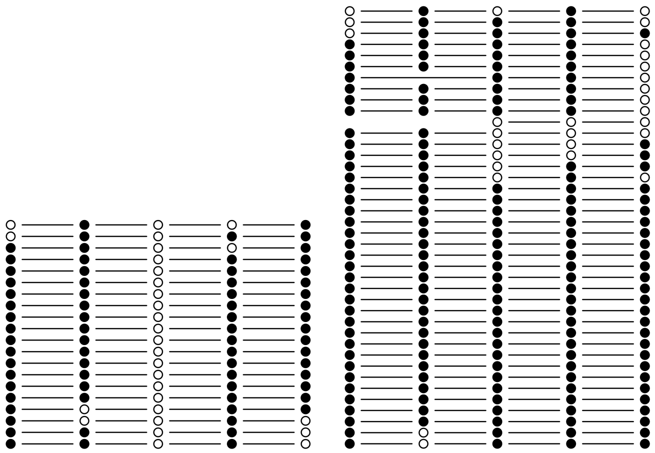

Figure 2: The two loci that are extracted from the observed data in Figure 1.

B MAP estimate for ϵ

Given $\mathbf{X}$, $\mathbf{w}$, Q and uniform priors $p(\mathbf{X}|Q)$ and $p(\mathbf{w}|Q)$ the MAP estimate for the hyperparameter ϵ is given by maximising the log of the posterior (2):

$$\begin{aligned}\mathcal{L}(\epsilon) &= \log p(\mathbf{Y}|\mathbf{X}, \mathbf{w}, \epsilon, Q) \\ &= \log \prod_{i=1}^{N} \prod_{\mu=1}^{d} p(y_{i\mu}|x_{w_i\mu}, \epsilon, Q) \\ &= \alpha_0 \log \epsilon + \alpha_1 \log(1-\epsilon). \end{aligned} \tag{1}$$

Note that on the second line that if read $\mathbf{y}_i$ originates from epiallele q then $w_i = q$. Recall that $\alpha_1 = \sum_{i,\mu} \delta_{y_{i\mu}, x_{w_i\mu}}$ and $\alpha_0 = \sum_{i,\mu} 1 - \delta_{y_{i\mu}, x_{w_i\mu}}$ denote the total number of matches and mismatches between the observed reads $\mathbf{y}$ and the corresponding epialleles $\mathbf{x}$ at this particular loci. Solving $\mathrm{d}\mathcal{L}/\mathrm{d}\epsilon = 0$ yields $\epsilon = \alpha_0/(\alpha_0 + \alpha_1)$.

C Simulation results

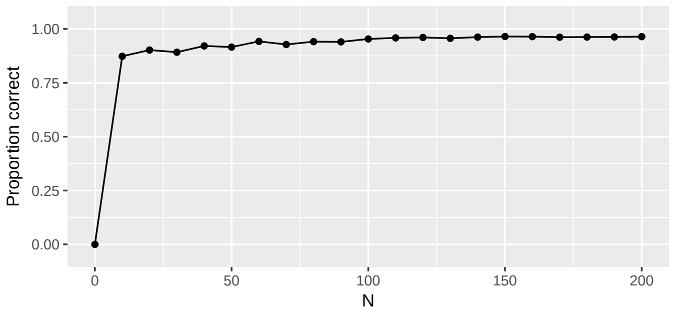

Figure 3: Proportion of observed reads attributed to the correct underlying epiallele as a function of N (the number of sequencing reads at the simulated locus). Parameters were fixed to $\epsilon = 0.05$, $d = 6$ and $Q = 3$.

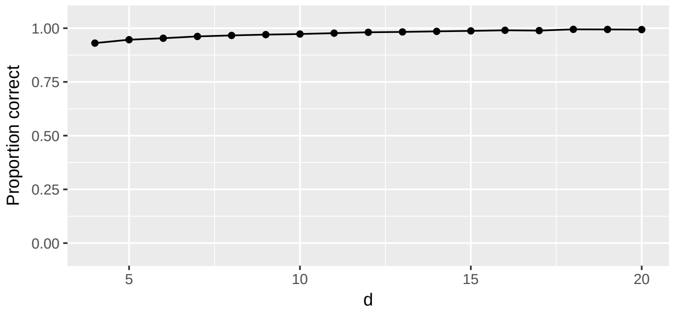

Figure 4: Proportion of observed reads attributed to the correct underlying epiallele as a function of d (the number of CpGs at the simulated locus). Parameters were fixed to $N = 100$, $\epsilon = 0.05$ and $Q = 3$.

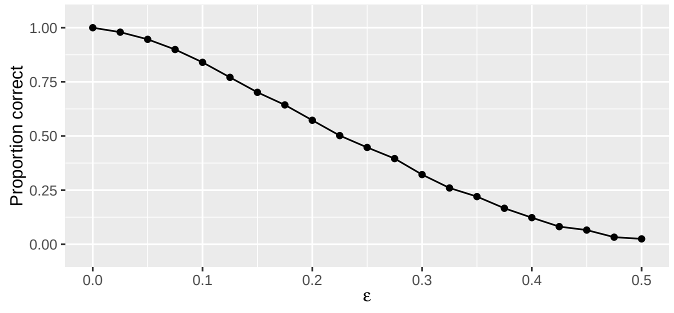

Figure 5: Proportion of observed reads attributed to the correct underlying epiallele as a function of ϵ (the noise level). Parameters were fixed to $N = 100$, $d = 6$ and $Q = 3$.

D Purity estimation

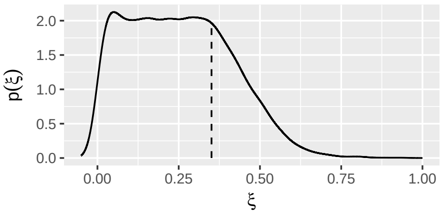

(a) R1

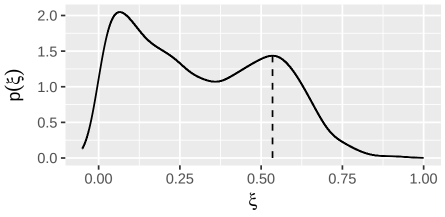

(b) R2

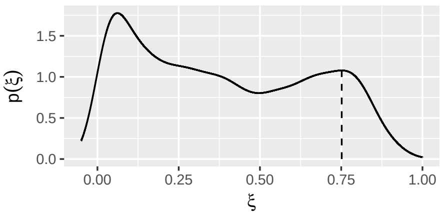

(c) R3

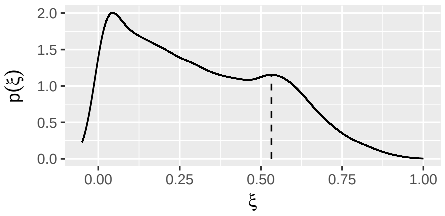

(d) R4

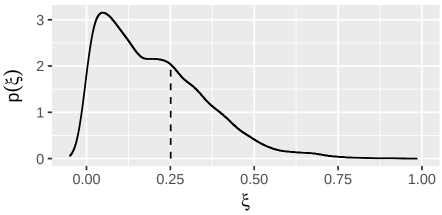

(e) R5

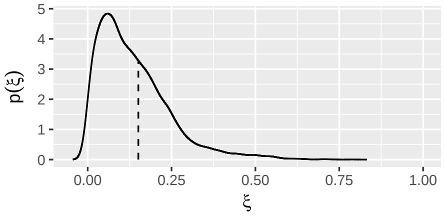

(f) R6

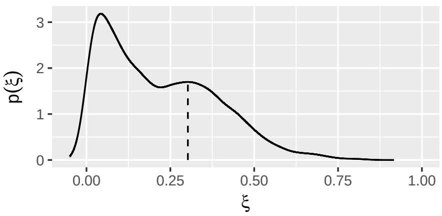

(g) R7

Figure 6: Empirical density plots of ξ, the proportion of epialleles at a locus that are different from normal tissue. The distribution of ξ will depend on the tumour purity since samples that are contaminated with less normal tissue will exhibit a greater deviance from the matched normal tissue epialleles. On this basis, the rightmost maxima (marked with a dashed vertical line) of the empirical densities are interpreted as a proxy for sample purity.

E Supplementary figures

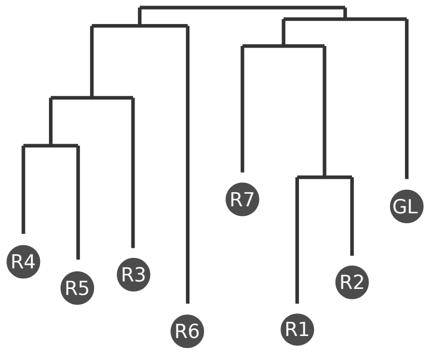

Figure 7: Phylogenetic tree generated from exome sequencing data from the same tumour that is studied in the main text. The exome data were generated and analysed independently as part of the Jamal-Hanjani et al., 2017 study. GL denotes germline.

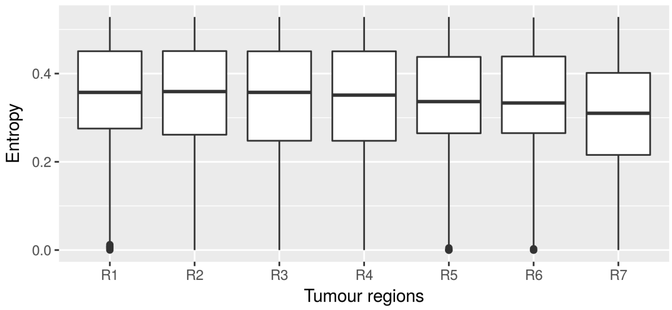

Figure 8: Box plots of the Shannon entropy of the epiallele distribution across the seven tumour regions (R1-R7) after decontamination of normal tissue.

F Comparison to alternative measures of disorder

As discussed in the main text the *epiallele entropy* provides a measure of epigenetic disorder within each tissue sample. In this section we compare our epiallele entropy approach to previously proposed measures of epigenetic disorder. In particular, we will compare the *epipolymorphism score* from Landan et al. (2012), the *methylation entropy* from Xie et al. (2011) and the *proportion of discordant reads* (PDR) approach from Landau et al. (2014).

Both the epipolymorphism score and the PDR score are restricted to loci with 4 CpGs. We have therefore restricted this comparison to only loci with 4 CpGs of which there 38,831 in total across all of our tissue samples. Furthermore, none of the comparators can handle missing data so any reads with a missing CpG measurement were discarded. As described in the main text for our

epiallele entropy approach we used the distribution of epialleles after marginalisation over the **w** parameter and any epialleles that had a frequency of less than 5% were discarded.

Box plots comparing the four different measures are shown in Figure 9. All four methods attribute lower disorder scores to the normal tissue. Low purity tumour regions such as R5, R6 and R7 are also given lower disorder scores by all four approaches. The greatest difference between normal and tumour tissue is observed using our epiallele entropy measure. Our method has the additional advantage that it can handle an arbitrary number of CpGs per locus and missing data.

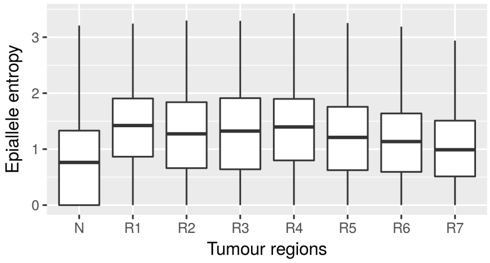

(a) Epiallele entropy

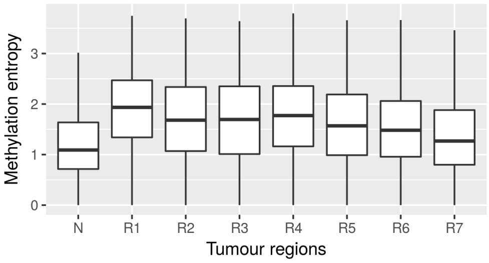

(b) Methylation entropy

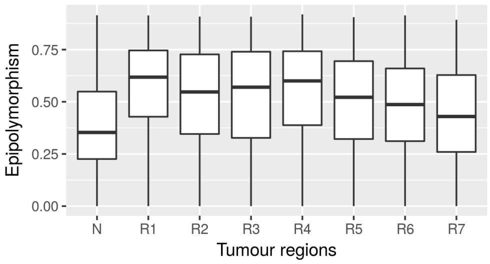

(c) Epipolymorphism score

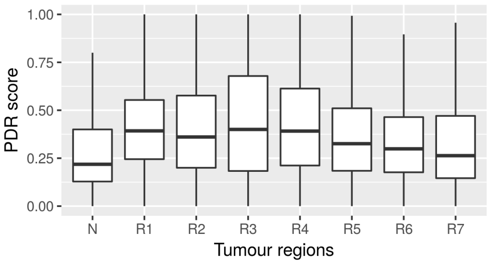

(d) PDR score

Figure 9: Box plots comparing different epigenetic disorder measures.

G Experimental datasets

The RRBS data are available at the European Nucleotide Archive under accession numbers ERS1546024, ERS1546025 and ERS1546026.

References

G. Landan et al. Epigenetic polymorphism and the stochastic formation of differentially methylated regions in normal and cancerous tissues. *Nat. Genet.*, 44(11):1207–1214, 2012.

D. Landau et al. Locally disordered methylation forms the basis of intratumor methylome variation in chronic lymphocytic leukemia. *Cancer Cell*, 26(6):813–825, 2014.

H. Xie et al. Genome-wide quantitative assessment of variation in DNA methylation patterns. *Nucleic Acids Res.*, 39(10):4099–4108, 2011.

H TRACERx consortium members

The TRACERx study (Clinicaltrials.gov no: NCT01888601) is sponsored by University College London (UCL/12/0279) and has been approved by an independent Research Ethics Committee (13/LO/1546). TRACER is funded by Cancer Research UK (grant number C11496/A17786) and coordinated through the Cancer Research UK & UCL Cancer Trials Centre.

Consortium members

Charles Swanton[1,2,5], Mariam Jamal-Hanjani[1], Selvaraju Veeriah[1], Seema Shafi[1], Justyna Czyzewska-Khan[1], Diana Johnson[1], Joanne Laycock[1], Leticia Bosshard-Carter[1], Gerald Goh[1], Rachel Rosenthal[1], Pat Gorman[1], Nirupa Murugaesu[1], Robert E Hynds[1,3], Gareth Wilson[1,2], Nicolai J Birkbak[1,2], Thomas B K Watkins[2], Nicholas McGranahan[1,2], Stuart Horswell[2], Richard Mitter[2], Mickael Escudero[2], Aengus Stewart[2], Peter Van Loo[2], Andrew Rowan[2], Hang Xu[2], Samra Turajlic[2,4], Crispin Hiley[2], Christopher Abbosh[1], Jacki Goldman[2], Richard Kevin Stone[2], Tamara Denner[2], Nik Matthews[2], Greg Elgar[2], Sophia Ward[2], Jennifer Biggs[2], Marta Costa[2], Sharmin Begum[2], Ben Phillimore[2], Tim Chambers[2], Emma Nye[2], Sofia Graca[2], Maise Al Bakir[2], Kroopa Joshi[1], Andrew Furness[1], Assma Ben Aissa[1], Yien Ning Sophia Wong[1], Andy Georgiou[1],Sergio Quezada[1], John A Hartley[1], Helen L Lowe[1], Javier Herrero[1], David Lawrence[5], Martin Hayward[5], Nikolaos Panagiotopoulos[5], Shyam Kolvekar[5], Mary Falzon[5], Elaine Borg[5], Teresa Marafioti[5], Celia Simeon[5], Gemma Hector[5], Amy Smith[5], Marie Aranda[5], Marco Novelli[5], Dahmane Oukrif[5], Sam M Janes[5], Ricky Thakrar[5], Martin Forster[5], Tanya Ahmad[5], Siow Ming Lee[5], Dionysis Papadatos-Pastos[5], Dawn Carnell[5], Ruheena Mendes[5], Jeremy George[5], Neal Navani[5], Asia Ahmed[5], Magali Taylor[5], Junaid Choudhary[5], Yvonne Summers[6], Raffaele Califano[6], Paul Taylor[6], Rajesh Shah[6], Piotr Krysiak[6], Kendadai Rammohan[6], Eustace Fontaine[6], Richard Booton[6], Matthew Evison[6], Phil Crosbie[6], Stuart Moss[6], Faiza Idries[6], Leena Joseph[6], Paul Bishop[6], Anshuman Chaturved[6], Anne Marie Quinn[6], Helen Doran[6], Angela leek[7], Phil Harrison[7], Katrina Moore[7], Rachael Waddington[7], Juliette Novasio[7], Fiona Blackhall[8], Jane Rogan[7], Elaine Smith[6], Caroline Dive[9], Jonathan Tugwood[9], Ged Brady[9], Dominic G Rothwell[9], Francesca Chemi[9], Jackie Pierce[9], Sakshi Gulati[9], Babu Naidu[10], Gerald Langman[10], Simon Trotter[10], Mary Bellamy[10], Hollie Bancroft[10], Amy Kerr[10], Salma Kadiri, [10], Joanne Webb[10], Gary Middleton[10], Madava Djearaman[10], Dean Fennell[11], Jacqui A Shaw[11], John Le Quesne[11], David Moore[11], Apostolos Nakas[12], Sridhar Rathinam[12], William Monteiro[13], Hilary Marshall[13], Louise Nelson[12], Jonathan Bennett[12], Joan Riley[12], Lindsay Primrose[12], Luke Martinson[12], Girija Anand[14], Sajid Khan[15], Anita Amadi[16], Marianne Nicolson[17], Keith Kerr[17], Shirley Palmer[17], Hardy Remmen[17], Joy Miller[17], Keith Buchan[17], Mahendran Chetty[17], Lesley Gomersall[17], Jason Lester[18], Alison Edwards[18], Fiona Morgan[19], Haydn Adams[19], Helen Davies[19], Malgorzata Kornaszewska[20], Richard Attanoos[21], Sara Lock[22], Azmina Verjee[22], Mairead MacKenzie[23], Maggie Wilcox[23], Harriet Bell[24], Natasha Iles[24], Allan Hackshaw[24], Yenting Ngai[24], Sean Smith[24], Nicole Gower[24], Christian Ottensmeier[25], Serena Chee[25], Benjamin Johnson[25], Aiman Alzetani[25], Emily Shaw[25], Eric Lim[26], Paulo De Sousa[26], Monica Tavares Barbosa[26], Alex Bowman[26], Simon Jordan[26], Alexandra Rice[26], Hilgardt Raubenheimer[26], Chiara Proli[26], Maria Elena Cufari[26], John Carlo Ronquillo[26], Angela Kwayie[26], Harshil Bhayani[26], Morag Hamilton[26], Yusura Bakar[26], Natalie Mensah[26], Lyn Ambrose[26], Anand Devaraj[26], Silviu Buderi[26], Jonathan Finch[26], Leire Azcarate[26], Hema Chavan[26], Sophie Green[26], Hillaria Mashinga[26], Andrew G Nicholson[26], [27], Kelvin Lau[28], Michael Sheaff[28], Peter Schmid[28], John Conibear[28], Veni Ezhil[29], Babikir Ismail[29], Melanie Irvin-sellers[29], Vineet Prakash[29], Peter

Russell[30], Teresa Light[30], Tracey Horey[30], Sarah Danson[31], Jonathan Bury[31], John Edwards[31], Jennifer Hill[31], Sue Matthews[31], Yota Kitsanta[31], Kim Suvarna[31], Patricia Fisher[31], Allah Dino Keerio[31], Michael Shackcloth[32], John Gosney[32], Pieter Postmus[32], Sarah Feeney[32], Julius Asante-Siaw[32], Tudor Constatin[33], Raheleh Salari[33], Nicole Sponer[33], Ashwini Naik[33], Bernhard Zimmermann[33], Hugo J.W.L. Aerts[34], Stefan Dentro[35], Christophe Dessimoz[36,37,38].

Affiliations

1. Cancer Research UK Lung Cancer Centre of Excellence, University College London Cancer Institute, United Kingdom
2. The Francis Crick Institute, United Kingdom
3. Lungs for Living, UCL Respiratory, University College London, United Kingdom
4. The Royal Marsden Hospital, United Kingdom
5. University College London Hospitals NHS Foundation Trust, United Kingdom
6. University Hospital of South Manchester, United Kingdom
7. Manchester Cancer Research Centre Biobank, United Kingdom
8. Christie NHS Foundation Trust, Manchester, United Kingdom
9. Cancer Research UK Manchester Institute, United Kingdom
10. Heart of England NHS Foundation Trust, Birmingham, United Kingdom
11. Cancer Studies and Molecular Medicine, University of Leicester, United kingdom
12. Leicester University Hospitals, United Kingdom
13. National Institute for Health Research Leicester Respiratory Biomedical, Research Unit, United Kingdom
14. North Middlesex Hospital, United Kingdom
15. Royal Free Hospital, United Kingdom
16. Barnet Hospital, United Kingdom
17. Aberdeen Royal Infirmary, United Kingdom
18. Velindre Cancer Centre, Cardiff, Wales, United Kingdom
19. Cardiff & Vale University Health Board, Cardiff, Wales, United Kingdom
20. University Hospital Of Wales Heath Park, Cardiff, Wales, United Kingdom
21. Department of Pathology, University Hospital of Wales and Cardiff University, Heath Park, Cardiff, Wales, United Kingdom
22. The Whittington Hospital NHS Trust, United Kingdom
23. Independent Cancer Patients Voice, United Kingdom
24. Cancer Research UK & UCL Cancer Trials Centre, United Kingdom
25. University Hospital Southampton NHS Foundation Trust, United Kingdom
26. Royal Brompton and Harefield NHS Foundation Trust, United Kingdom
27. National Heart and Lung Institute, Imperial College, United Kingdom
28. Barts Health NHS Trust, United Kingdom
29. Ashford and St. Peter's Hospitals NHS Foundation Trust, United Kingdom
30. The Princess Alexandra Hospital NHS Trust, United Kingdom
31. Sheffield Teaching Hospitals NHS Foundation Trust, United Kingdom
32. Liverpool Heart and Chest Hospital NHS Foundation Trust, United Kingdom
33. Natera Inc., 201 Industrial Road, Suite 410, San Carlos, CA 94070
34. Dana-Farber Cancer Institute, Brigham & Women's Hospital, Harvard Medical School, 450 Brookline Ave, JF518, Boston, MA 02115-5450, USA

35. Wellcome Trust Sanger Institute, Hinxton, CB10 1SA, United Kingdom
36. Bioinformatics Group, Department of Computer Science, University College London
37. University of Lausanne
38. Swiss Institute of Bioinformatics
